# Supplementary material for: Identification of photoperiod-induced specific miRNAs in the adrenal glands of Sunite sheep (Ovis aries)
Source: Front Vet Sci. 2022 Jul 22;9:888207. doi: 10.3389/fvets.2022.888207 (PMC9354845; doi:10.3389/fvets.2022.888207)
Supplement: Supplementary file 1 [file Data_Sheet_1.ZIP › Supplementary file/miRDeep2 file.zip!/miRDeep2 file/Novel_Structure/LP42A2/Novel_131.pdf]

Provisional ID	:	NC_019468.2_20305
Score total	:	2
Score for star read(s)	:	-1.3
Score for read counts	:	0
Score for mfe	:	1.7
Score for randfold	:	1.6
Score for cons. seed	:	
Total read count	:	40
Mature read count	:	40
Loop read count	:	0
Star read count	:	0

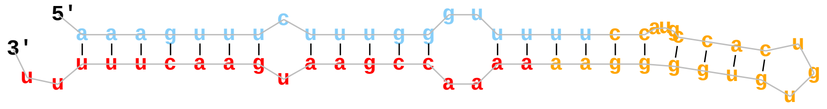

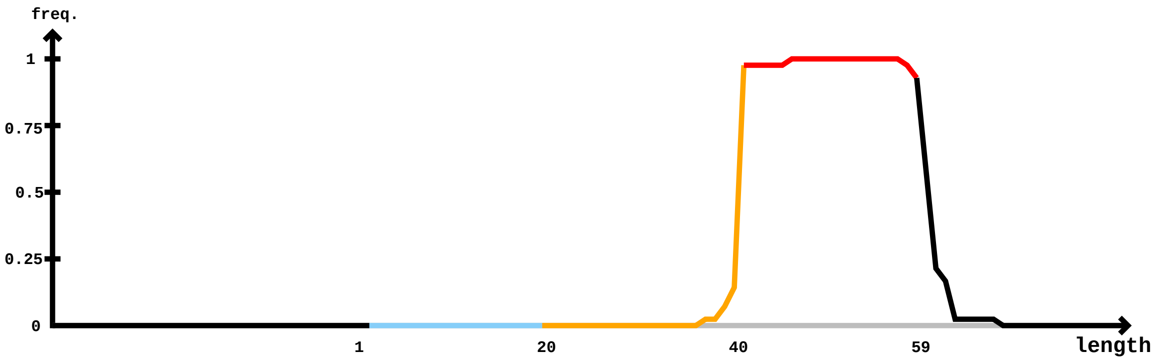

Star Mature

```
5'- caguggaaguaauuuguauuggguugaccaaaaaguuucuuuggguuuuuccaugccacugugugggg aaaaaaccgaaugaacuuuuuggccaacacaauauuuucuuu   -3'    exp
    ....(((((......((((((.((((.(((((((((((.(((((..((((((...((((...))))))))))..))))).))))))))))).)))).)))))))))))..    reads     mm     sample
    ..................................................................ggaaaaaCccgaaugaacuuu.......................    1         1      seq
    ....................................................................aaaaaCccgaaugaacuuuu......................    2         1      seq
    .....................................................................aaaaCccgaaugaacuuuuu.....................    1         1      seq
    .....................................................................aaaaCccgaaugaacuuuuugg...................    2         1      seq
    ......................................................................aaaCccgaaugaacuuuuu.....................    28        1      seq
    ......................................................................aaaaccgaaugaacuuuuug....................    1         0      seq
    ......................................................................aaaCccgaaugaacuuuuuggc..................    6         1      seq
    ...........................................................................cAaaugaacuuuuuggccaaca.............    1         1      seq
```
